# Serum Lowers Bioactivity and Uptake of Synthetic Amorphous Silica by Alveolar Macrophages in a Particle Specific Manner

**DOI:** 10.3390/nano11030628

**Published:** 2021-03-03

**Authors:** Martin Wiemann, Antje Vennemann, Cornel Venzago, Gottlieb-Georg Lindner, Tobias B. Schuster, Nils Krueger

**Affiliations:** 1 IBE R&D Institute for Lung Health gGmbH, Mendelstr. 11, 48149 Münster, Germany; vennemann@ibe-ms.de; 2 Evonik Operations GmbH, Rodenbacher Chaussee 4, 63457 Hanau-Wolfgang, Germany; cornel.venzago@evonik.com (C.V.); tobias.schuster@evonik.com (T.B.S.); nils.krueger@evonik.com (N.K.); 3 Evonik Operations GmbH, Brühler Straße 2, 50389 Wesseling, Germany; gottlieb-georg.lindner@evonik.com

**Keywords:** nanomaterials, synthetic amorphous silica, in vitro testing, NR8383 alveolar macrophage, ICP-MS analysis of cell bound SiO_2_

## Abstract

Various cell types are compromised by synthetic amorphous silica (SAS) if they are exposed to SAS under protein-free conditions in vitro. Addition of serum protein can mitigate most SAS effects, but it is not clear whether this is solely caused by protein corona formation and/or altered particle uptake. Because sensitive and reliable mass spectrometric measurements of SiO_2_ NP are cumbersome, quantitative uptake studies of SAS at the cellular level are largely missing. In this study, we combined the comparison of SAS effects on alveolar macrophages in the presence and absence of foetal calf serum with mass spectrometric measurement of ^28^Si in alkaline cell lysates. Effects on the release of lactate dehydrogenase, glucuronidase, TNFα and H_2_O_2_ of precipitated (SIPERNAT^®^ 50, SIPERNAT^®^ 160) and fumed SAS (AEROSIL^®^ OX50, AEROSIL^®^ 380 F) were lowered close to control level by foetal calf serum (FCS) added to the medium. Using a quantitative high resolution ICP-MS measurement combined with electron microscopy, we found that FCS reduced the uptake of particle mass by 9.9% (SIPERNAT^®^ 50) up to 83.8% (AEROSIL^®^ OX50). Additionally, larger particle agglomerates were less frequent in cells in the presence of FCS. Plotting values for lactate dehydrogenase (LDH), glucuronidase (GLU) or tumour necrosis factor alpha (TNFα) against the mean cellular dose showed the reduction of bioactivity with a particle sedimentation bias. As a whole, the mitigating effects of FCS on precipitated and fumed SAS on alveolar macrophages are caused by a reduction of bioactivity and by a lowered internalization, and both effects occur in a particle specific manner. The method to quantify nanosized SiO_2_ in cells is a valuable tool for future in vitro studies.

## 1. Introduction

Synthetic amorphous silica (SAS) form a major group of industrially relevant nanomaterials (NMs) [1,2,3]. They are produced either from aqueous solutions of sodium silicate to form colloidal silica, silica gels, and precipitated silica, or may be synthesized from the gaseous phase of SiCl_4_ to form fumed (pyrogenic) silica [3,4]. Due to the production process, pyrogenic and precipitated silica form indivisible aggregates, which have no physical boundaries among their primary structures. These aggregates have external dimensions that are highly variable in nature with some particles being in the nano-size range [3,5]. Since most SAS materials come as dry powders, a non-intentional uptake into the body may occur via inhalation [6,7]. Animal studies have shown that SAS can induce transient inflammatory responses in the rat lung [6,7,8,9,10] whereas fibrogenic or genotoxic effects, as induced by crystalline silica, such as quartz or cristobalite, were not induced even at high lung burden [11,12].

To describe and predict the bioactivity of the multitude of SAS, in vitro assays using different cell types and incubation conditions have been published [13,14,15,16,17]. Recently, the effects of SAS from all major production processes were tested using rat alveolar macrophages (NR8383) in vitro [18]. A major finding was that SAS, irrespective of the production method, elicit highly uniform responses, e.g., with respect to the release of lactate dehydrogenase (LDH), glucuronidase (GLU), tumour necrosis factor alpha (TNFα) or induction of H_2_O_2_ release [18]. In these experiments, cells were exposed to SAS under protein-free standard conditions, i.e., in the absence of serum proteins. This is a well-established testing procedure reflecting the circumstance that particles entering into lung alveoli are primarily protein-free entities. Furthermore, the protein-free in vitro conditions allow to study particle effects linked, e.g., to surface properties of nanoparticles [19]. However, for most SAS, the half maximal effective concentration (EC50) for several endpoints is comparatively low under protein-free conditions (e.g., 10–20 µg/mL for the release of LDH) [18], a finding which does not correspond to the in vivo effects of SAS especially when effects of amorphous and crystalline silica are compared [12]. On the other hand, the bioactivity of SAS in vitro is strongly reduced when particles (i) are administered in the presence of serum [20], (ii) are protein-treated prior to exposure [21,22,23,24] or (iii) are dispersed by extensive ultrasonic energy in the presence of low concentrations of albumin [25]. All aforementioned protein-treatments inevitably lead to the formation of a protein corona [26,27]. However, they also influence particle dispersion, gravitational settling and uptake by cells. Studies with fluorescent silica probes showed a decreased gravitational settling in the presence of protein, whereas larger precipitates of SAS settled onto the cells in the absence of protein [21,22,28]. The direct contact with the cell membrane and/or the uptake of particles is a prerequisite at least for poorly soluble particles to elicit cellular effects. Therefore, a lower rate of gravitational settling can reduce the effects of nanoparticles under submersed in vitro conditions [29]. Interestingly, the addition of protein has no uniform effect on the uptake of SAS particles and this appears to be especially relevant for macrophages. In RAW264.7 macrophages, addition of FCS enabled the uptake of SAS but mitigated cytotoxic effects [21]. In contrast, albumin-coating of NM-200, a precipitated SAS and of the pyrogenic NM-203 led to more cytotoxicity in THP-1 cells, but lowered the cytotoxicity in RAW264.7 cells [25]. Another unexpected result was provided by Binnemars–Postma and co-workers, who showed that human M1 macrophages ingested more silica nanoparticles in the absence of serum, whereas the presence of serum increased the uptake of SAS by M2 macrophage [30]. Although not yet fully elucidated, these discrepancies may be due to the presence of surface receptors involved in particle uptake, such as the scavenger receptor expressed in RAW264.7 cells [31]. Together, these results show that the effects of FCS on bioactivity and uptake of SAS are cell-type specific and need to be explored more thoroughly, especially when different types of macrophages are compared.

In the present paper, we aim to close this gap for NR8383 alveolar macrophages from rat lung using fumed and precipitated SAS with small and large specific surface areas, which were chosen to represent the large number of different SAS on the market. The well-established NR8383 cell line is widely used to analyse the bioactivity of particles in the lung: in the so-called macrophage model, NR8383 cells are exposed to settling particles under submersed conditions. The particles’ bioactivity in the lung is then predicted from a set of assays carried out with the cell culture supernatant [19,32,33,34,35,36]. However, because SAS particles are small, their gravitational settling followed by cellular uptake may be incomplete counteracting a reliable dosimetry. Although elaborated models may predict the fraction of gravitationally settled of SAS in a time-dependent manner [37], there is still a need for quantitative measurements of SAS in cells. The quantification of SAS in cells requires a sensitive and valid analytical method. Since the mass spectrometric measurement of silicon suffers from N_2_ interference, an instrument with a high mass resolution is needed. Moreover, the conventional solubilisation of silica particles in organic matter with hydrofluoric acid (HF) is subject to critical handling guidelines and requires specialized labs and personnel [38]. Recently, Bossert et al. (2019) proposed a HF-free hot alkaline lysis followed by acid treatment to dissolve silica particles; silicon was then detected by ICP-OES or by a colorimetric method with a limit of detection (LOD) being in the range of 40–100 mg/L [39]. Of note, cells exposed in vitro to approximately 10 µg SAS per mL likely underscore this LOD by at least one order of magnitude, unless the cell number is scaled up to very high amounts. To meet more conventional dimensions of cell culture testing, i.e., testing of several million cells per well, here we present a sufficiently sensitive method for SAS quantification, combining alkaline dissolution, acid neutralization and high resolution ICP-MS. By this, the amount of cellular SAS was measured and compared with the subcellular distribution of SAS in NR8383 cells in the absence and presence of foetal calf serum (FCS). This way the effect of FCS, known to reduce the bioactivity of SAS on macrophages in vitro, could be analysed for the first time on the basis of quantitative uptake data. 

## 2. Materials and Methods

### 2.1. Materials

The materials were provided by Evonik Operations GmbH (Hanau-Wolfgang, Germany) as dry powders and had been extensively characterized in a previous publication [18]. The main data are summarized in Table 1.

### 2.2. Preparation of Particle Suspensions

The main goal of the study was to compare effects of SAS in the absence and presence of foetal calf serum, after particles had been dispersed according to a previously established protocol [18]. In brief, particles were suspended in sterile H_2_O (Aqua ad injectabilia, Braun Melsungen, Germany) at a concentration of 2 mg/mL. Suspensions were vortexed, stirred with a magnetic bar for 90 min and passed through a sterile polyamide gauze with a nominal pore width of 5 µm (Bückmann, Mönchengladbach, Germany) (see [4] for filtration characteristics). 5 mL of each filtrate was then transferred to a 20 mL glass vial and subjected to an ultrasonic dispersion energy 270 J/mL [4]. Dispersed masses amounted to 70–100% the original masses, as determined by gravimetric analysis. The stock aqueous stock suspensions of SIPERNAT^®^ 160, SIPERNAT^®^ 50, AEROSIL^®^ OX50 and AEROSIL^®^ 380 F were adjusted to 360 µg/mL, and stored at 4 °C for up to 4 weeks. Immediately before experiments, aqueous stock suspensions were mixed with an equal volume double concentrated KRPG buffer (see below) or F-12K media to obtain a physiologic medium composition.

### 2.3. Cultivation of NR8383 Macrophages and Cell Culture Assays

NR8383 cells (ATCC, Manassas, VA, USA; ATCC^®^ Number: CRL-2192TM) were maintained in F-12K cell culture medium (Sigma-Aldrich, Taufkirchen, Germany) supplemented with 15% foetal calf serum (FCS), 1% penicillin/streptomycin and 1% L-glutamine (all from PAN Biotech, Aidenbach, Gremany) under cell culture conditions (37 °C and 5% CO_2_) [32]. For the assay, 3 × 10^5^ cells were seeded per well of a 96-well plate, and covered with 200 µL F-12K cell culture medium plus 5% (*v*/*v*) FCS to foster cell adherence. The next day, the medium was replaced by serum-free or FCS (10%)-containing F-12K medium containing increasing concentrations of each material (11.25, 22.5, 45 or 90 µg/mL). After 16 h, supernatant was retrieved to determine LDH, GLU and TNFα. To measure the release of H_2_O_2_, materials were equivalently diluted in KRPG buffer (129 mM NaCl, 4.86 mM KCl, 1.22 mM CaCl_2_, 15.8 mM NaH_2_PO_4_, 5–10 mM glucose; pH 7.3–7.4). 

Assays were carried out as described [32]. In brief, H_2_O_2_ was quantified with the Amplex Red^®^ assay by photometrically measuring formed resorufin at 570 nm (reference value: 620 nm) with a plate reader (Tecan Infinite F200Pro, Tecan GmbH, Crailsheim, Germany); positive controls were run with 360 µg/mL zymosan (Sigma-Aldrich, Taufkirchen, Germany). Measurements were corrected for background absorbance of cell free-particle controls and converted into concentrations of H_2_O_2_ as described. LDH activity was measured with the Roche Cytotoxicity Kit (Sigma-Aldrich, Taufkirchen, Germany) according to the manufacturer’s protocol. GLU activity was detected with p-nitrophenyl-D-glucuronide dissolved in 0.2 M sodium acetate buffer (pH 5) containing 0.1% Triton X-100. Both the LDH- and GLU-based values were corrected for cell-free absorption and normalised to the positive control (0.1% Triton X-100 in F-12K) which was set to 100%. Tumour necrosis factor α (TNF α) was determined with a specific enzyme-linked immosorbent assay (ELISA) for rat TNFα (Quantikine ELISA Kit) according to the manufacturer’s protocol (Bio-Techne GmbH, Wiesbaden-Nordenstadt, Germany). The TNFα-forming capacity of NR8383 cells was tested with 0.5 µg/mL lipopolysaccharide (LPS, Sigma-Aldrich, Taufkirchen, Germany). Notably, aliquots for measuring LDH, GLU and TNFα were taken from the same well.

### 2.4. Particle Size Determination under Cell Culture Conditions with Particle Tracking Analysis 

The hydrodynamic diameter was determined by optical tracking analyses using a NanoSight LM10 instrument equipped with a violet laser (405 nm), an Andor CCD camera, and particle tracking software NTA3.0 (all from: Malvern Instruments GmbH, Herrenberg, Germany). Starting with the aqueous particle stock suspension, dilutions were prepared in KRPG and F-12K medium in the absence and presence of 10% FCS. Concentration was uniformly set to 90 µg/mL. Suspensions were incubated under cell culture conditions (37 °C, 5% CO_2_, 100% humidity) for 90 min (KRPG) and 16 h (F-12K), respectively. Suspensions were further diluted to obtain measurable concentrations, approximately in the range of 5 × 10^8^ particle/mL.

### 2.5. Electron Microscopy of NR8383 Macrophages

NR8383 cells were seeded onto small discs (diameter 6 mm) of Melinex film (Plano, Wetzlar, Germany) placed in the wells of a 96-well plate, subjected to particle treatment for 16 h as described in paragraph 2.4. Then, media were withdrawn and cells were immediately covered with 2.5% glutardialdehyde in 0.1 M sodium phosphate buffer (SPB, pH 7.3) for 60 min. Cells were washed three times with SPB, post-fixed in 1% OsO_4_, dehydrated in ethanol to the 70% step, and stained en bloc with uranium acetate (1%). Cells were dehydrated via ethanol/propylene oxide, and embedded in Epon 812 (Sigma Aldrich, Taufkirchen, Germany). Ultrathin sections (50–60 nm) were viewed with a Tecnai G2 electron microscope operated at 100 or 120 kV; images were taken with a Quemesa digital camera (Olympus Soft Imaging Solutions, Münster, Germany). 

### 2.6. Quantification of Cell-Associated SiO_2_ NP by High Resolution ICP-MS

To quantitate the amount of cell-associated SiO_2_ NP, 2.8 × 10^6^ NR8383 cells were seeded into each well of a 6-well plate and incubated in 6 mL F-12K medium containing 11.25 µg/mL of each SiO_2_ samples, both in the absence and presence of 10% FCS. After 16 h, the culture medium was completely withdrawn and replaced by 1 mL phosphate buffered saline (PBS). Cells were detached from the plates by vigorous pipetting and transferred into a 15 mL test tube (Falcon) pre-loaded with 6 mL fresh F-12K medium. Cells were spun down (200× *g*, 10 min), washed with 2 mL KRPG buffer, pelleted again (200× *g*, 10 min), and finally re-suspended in 130–230 µL KRPG buffer. A defined volume (90%) of this final suspension was dehydrated at 60 °C for 12 h for analysis. 

To measure the SiO_2_ content, each sample was dissolved with 50 µL of 20% NaOH and heated to 120 °C for 2 h. Lysates were diluted with 5 mL ultrapure H_2_O. In further dilution steps, 4% (*v*/*v*) of a HNO_3_ solution was added. Measurements of Si were carried out with a double focusing magnetic sector field ICP-MS instrument in the medium resolution mode (Element 2™, Thermo Fisher Scientific, Meerbusch, Germany) equipped with a quartz spray chamber and a quartz injection device (sample loop: 500 µL, ESI-Fastvalve). ^115^In was used as an internal standard. To calibrate the ICP-MS signal for ^28^Si, a Si standard stock solution of 100 mg/L (Labkings, Hilversum, The Netherlands) was diluted with 4% (*v*/*v*) of a HNO_3_-solution and an equivalent amount of NaOH as mentioned above to obtain a calibration range of 0.5–100 μg/L. Final SiO_2_ concentrations were obtained by multiplying measured Si concentrations with the stoichiometric factor of 2.1393. 

### 2.7. Statistical Evaluation

For in vitro testing, i.e., effects on LDH, GLU, H_2_O_2_, and TNFα, three independent repetitions were carried out; data were expressed as mean ± standard deviation (SD). To find significant differences, values from each concentration step were compared to the respective vehicle-treated control using 2-way analysis of variance (ANOVA) with Dunnett’s multiple comparisons test. Calculations were carried out with GraphPad Prism software. A value of *p* ≤ 0.05 was considered significant. Calculation of hydrodynamic diameters were carried out with NTA 3.0 software. 

## 3. Results

### 3.1. Particle Characterization

Two precipitated and two fumed SAS were selected for this study, whose major physical-chemical properties are shown in Table 1. A smaller and a larger particle type was selected for each group, also reflected by the BET values which, overall, span more than one order of magnitude. Acidity of the SAS powders was low but more pronounced for both AEROSIL^®^s. The SEARs No., which is a measure for the number of silanol groups at the particles’ surface, was similar for three SAS but lower for AEROSIL^®^ OX50. Solubility was very similar (112.1 to 117.9 mg/L) but slightly higher for AEROSIL^®^ 380 F.

**Figure 1:**
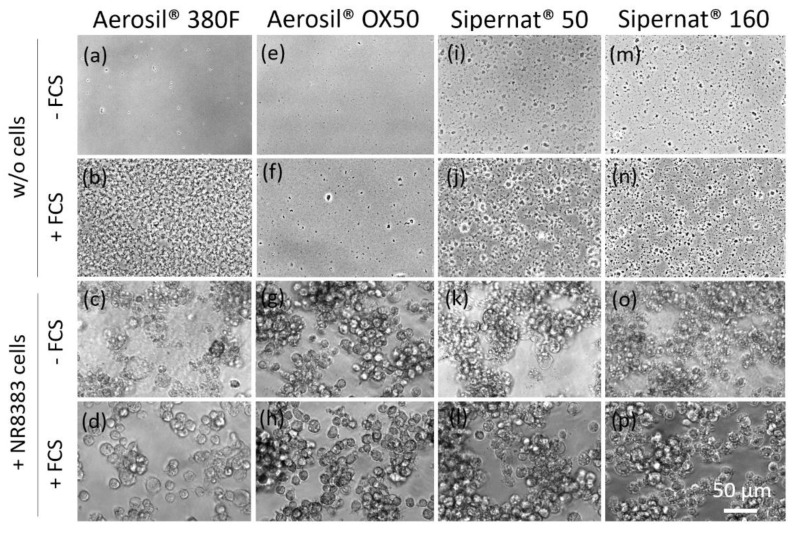
Sedimentation and uptake of precipitates by NR8383 cells which were formed in the presence of AEROSIL^®^ 380 F (**a**–**d**), AEROSIL^®^ OX50 (**e**–**h**), SIPERNAT^®^ 50 (**i**–**l**) and SIPERNAT^®^ 160 (**m**–**p**). Micrographs were taken 16 h after administration of particles (180 µg/mL) in the absence (w/o cells) and presence of cells (+NR8383). Foetal calf serum (FCS) led to the formation of agglomerates. In the absence of serum, many cells appeared deteriorated.

The addition of FCS to a SAS dispersion is likely to change the particles’ surface charge and agglomeration behaviour which is highly relevant for in vitro testing. Therefore, the hydrodynamic diameter (HD) of all four SAS was measured with particle tracking analysis (PTA) in H_2_O, and in the absence and presence of 10% foetal calf serum (FCS) in KRPG and F-12K medium, to mimic testing conditions (Table S1). In the case of SIPERNAT^®^ 160 and SIPERNAT^®^ 50, the HD (mode values) obtained in KRPG and F-12K were only slightly increased (<15%) compared to H_2_O; HD values also hardly increased upon of 10% FCS. AEROSIL^®^ 380 F particles were too small to be measured in H_2_O but measurable agglomerates were found in F-12K medium especially in the presence of FCS. The size of AEROSIL^®^ OX50 agglomerates increased in KRPG only. Overall, the effects of FCS on the hydrodynamic diameter of the particle fraction measurable by PTA were low. Nevertheless, the addition of FCS led to the formation of agglomerates visible by light microscopy at the bottom of the culture vessels (Figure 1). This effect was pronounced for AEROSIL^®^ 380 F (Figure 1a,b), but low for AEROSIL^®^ OX50 (Figure 1e,f), SIPERNAT^®^ 160 (Figure 1i,j) and SIPERNAT^®^ 50 (Figure 1m,n).

### 3.2. Quantification of Particle Uptake by NR8383 Cells

To measure the cell-associated SAS fraction which comprises internalized plus surface-bound particulate matter, we administered the lowest concentration of SAS particles (11.25 µg/mL) to a defined number of cells for 16 h (2.8 × 10^6^ per well). As expected, this treatment led to a low amount of dead cells in the absence (4.2–10.6%) and nearly no dead cells in the presence of serum (1.5–2.5%, see Table S2). Table 2 shows the cell-associated SiO_2_ masses measured by ICP-MS: Control cells contained low, though measurable amounts of ^28^Si. All SAS-treated cells showed a cell-associated SiO_2_ mass above background level, which could not be lowered, e.g., by avoiding SiO_2_ containing cell culture material (data not shown). In the presence of 10% FCS, values for the precipitated SAS SIPERNAT^®^ 50 and SIPERNAT^®^ 160 were higher than those for the fumed SAS AEROSIL^®^ OX50 and AEROSIL^®^ 380 F and this difference was not obvious under FCS-free conditions (Table 2). The cell-associated amount of SiO_2_ was reduced in the presence of 10% FCS-free conditions. The effect was pronounced for AEROSIL^®^ 380 F (−69.5%), AEROSIL^®^ OX50 (−83.8%), and SIPERNAT^®^ 160 (−62.3%), but comparatively small for SIPERNAT^®^ 50 (−9.9%).

### 3.3. In Vitro Toxicity Determination of SAS and Electron Microscopic Study

In vitro toxicity of SAS was measured in the absence and presence of FCS with the well-established alveolar macrophage assay. The activity of lactate dehydrogenase (LDH) and glucuronidase (GLU), as well as the concentration of tumour necrosis factor α (TNFα), were determined in the cell culture supernatant. Corundum and quartz DQ12 particles were included as negative and positive particle controls, respectively. Numerical results are shown in Table S3. The subcellular distribution of particles was investigated by transmission electron microscopy (TEM) of cells treated with the lowermost particle concentration of the study (11.25 µg/mL) and matched the particle concentration used above for the quantification experiments. 

#### 3.3.1. AEROSIL^®^ 380 F and AEROSIL^®^ OX50

**Figure 2:**
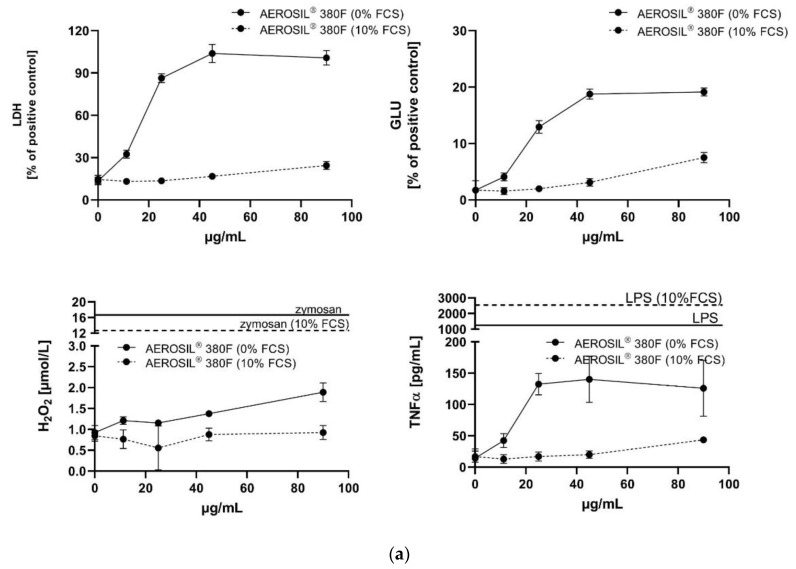

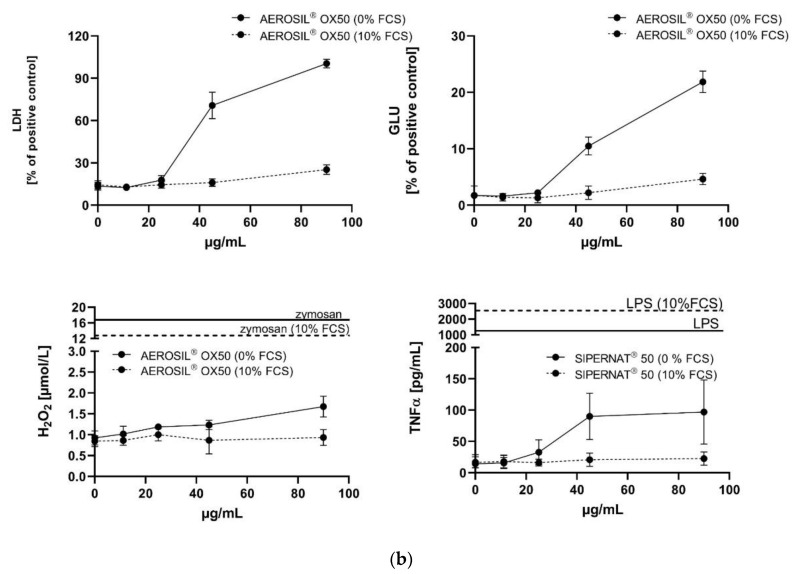
In vitro response of NR8383 alveolar macrophages to AEROSIL^®^ 380 F and to AEROSIL^®^ OX50 in the absence or in the presence of 10% FCS (dashed lines). Lactate dehydrogenase activity (LDH), glucuronidase activity (GLU), H_2_O_2_ concentration, and tumour necrosis factor alpha (TNFα) were measured in the supernatant from NR8383 cells exposed to AEROSIL^®^ 380 F (**a**) or AEROSIL^®^ OX50 (**b**). Effects of zymosan and lipopolysaccharide (LPS) on the formation of H_2_O_2_ and TNFα, respectively, are indicated by vertical lines.

Both AEROSIL^®^s showed a high biologic activity under FCS-free conditions (Figure 2a,b), indicated by the dose-dependent release of LDH, GLU and TNFα. As for most SAS particles the release of H_2_O_2_ was low and became significant at the highest doses only. In the presence of 10% FCS, all aforementioned responses were abolished (H_2_O_2_) or drastically lowered, as indicated by the flattened curves for the release of LDH, GLU and TNFα. The degree of reduction and the shift in the low observed adverse effect concentration (LOAEC) are provided in Table 3. 

**Figure 3:**
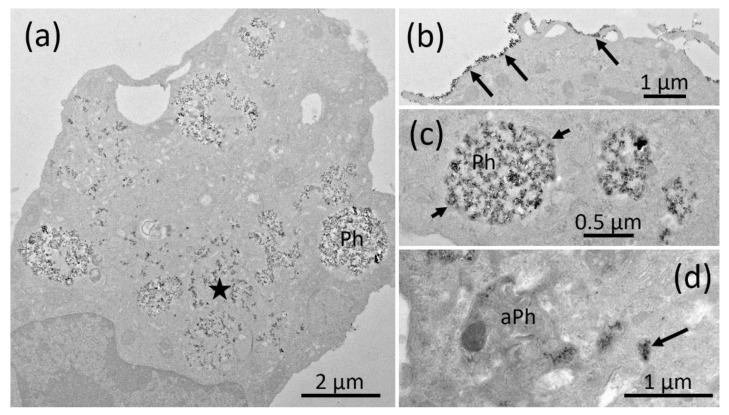
Electron microscopy of NR8383 cells laden with AEROSIL^®^ 380 F (11.25 µg/mL) in the absence of FCS for 16 h. (**a**) Overview of a cell containing particle filled phagosomes (Ph) and branched particle assemblies in distinct regions of the cytoplasm (asterisk). The cytoplasm of this cell appears condensed. (**b**) Particles adhering to a section of the outer cell membrane (arrows). (**c**) Particle filled phagosomes; arrows point to the enclosing membrane. (**d**) An autophagosome (aPh) filled with condensed matter together with several electron dense lysosomes (arrow); both compartments contain small amounts of the typical, small electron dense AEROSIL^®^ 380 F particles.

**Figure 4:**
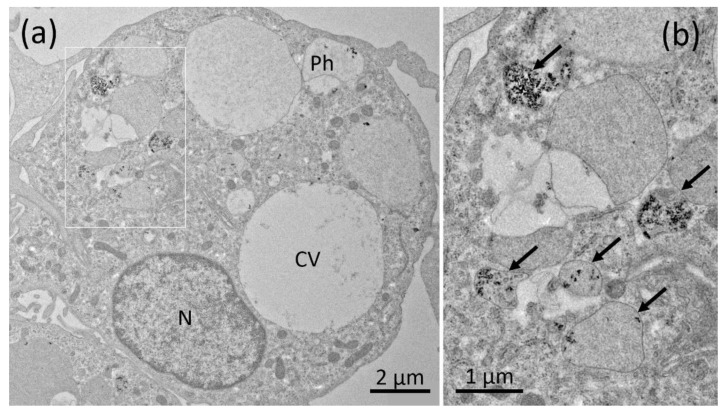
Electron microscopy of NR8383 cells laden with AEROSIL^®^ 380 F (11.25 µg/mL) in the presence of FCS for 16 h. (**a**) Aspect from a cell with typical phagosomes (Ph) mainly filled with fine granular material, particle laden lysosomes and a clear vacuole (CV). (**b**) Boxed area from (**a**) showing several particle-laden lysosomes (arrows).

The TEM investigation of NR8383 cells laden with AEROSIL^®^ 380 F in the absence of FCS cells is shown in Figure 3a–d. Although minor portions of the material were regularly found at the outer cell membrane (Figure 3b), larger assemblies occurred within phagosomes (Figure 3a,c). Small particle deposits were found in lysosomes (Figure 3d) and autophagosomes, together with condensed cellular material (Figure 3d). Of note, small and often branched aggregates/agglomerates of AEROSIL^®^ 380 F particles occurred in the cytoplasm; neither mitochondria nor the cell nucleus were found to contain particles. In the *presence of FCS*, particles (aggregates/agglomerates) of AEROSIL^®^ 380 F were not found at the outer cell membrane or within the cytoplasm. Additionally, heavily laden phagosomes were not found. Instead, cells contained large phagosomes filled with fine granular material of low-to-medium electron density (Figure 4a). Particles were mainly found in lysosomes (Figure 4b).

**Figure 5:**
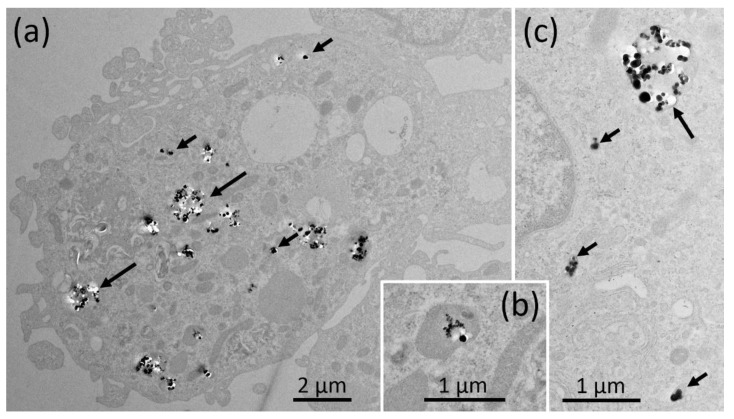
Electron microscopy of NR8383 cells laden with AEROSIL^®^ OX50 F in the absence of FCS for 16 h. Electron lucent areas close to particles are interpreted as cutting artefacts. (**a**) Overview of a cells with particle-containing phagosomes (large arrows) and smaller endosomes (small arrows). (**b**) shows a typical particle-laden; (**c**) shows a higher magnification of a phagosome (large arrow) and three small endosomes (small arrows) and arrows point to membrane continuities.

**Figure 6:**
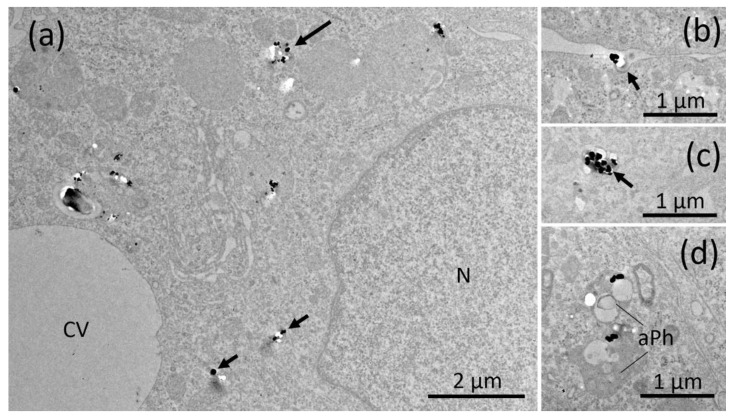
Electron microscopy of NR8383 cells laden with AEROSIL^®^ OX50 F in the presence of FCS for 16 h. Electron lucent areas close to particles are interpreted as cutting artefacts. (**a**) Overview of a cell with particle-containing phagosomes (large arrows) and smaller endosomes (small arrows). (**b**) A membrane invagination (arrow) underneath a particle attached to the cell membrane, interpreted as an early uptake figure. (**c**) A small particle-filled endosome; (**d**) shows two particle-containing autophagosomes (aPh).

AEROSIL^®^ OX50 was found as single particles or small groups thereof within endosomes, most likely presenting lysosomes, and phagosomes (Figure 5a–c). Although the presence of FCS did not lead to a major change of this pattern, smaller aggregates appeared to be more frequent (Figure 6a–c). Typical uptake-figures (Figure 6b) showed single particles close to a membrane invagination, suggesting that particles enter into cells via small endosomes. The material was also found in autophagosomes.

#### 3.3.2. SIPERNAT^®^ 50 and SIPERNAT^®^ 160

**Figure 7:**
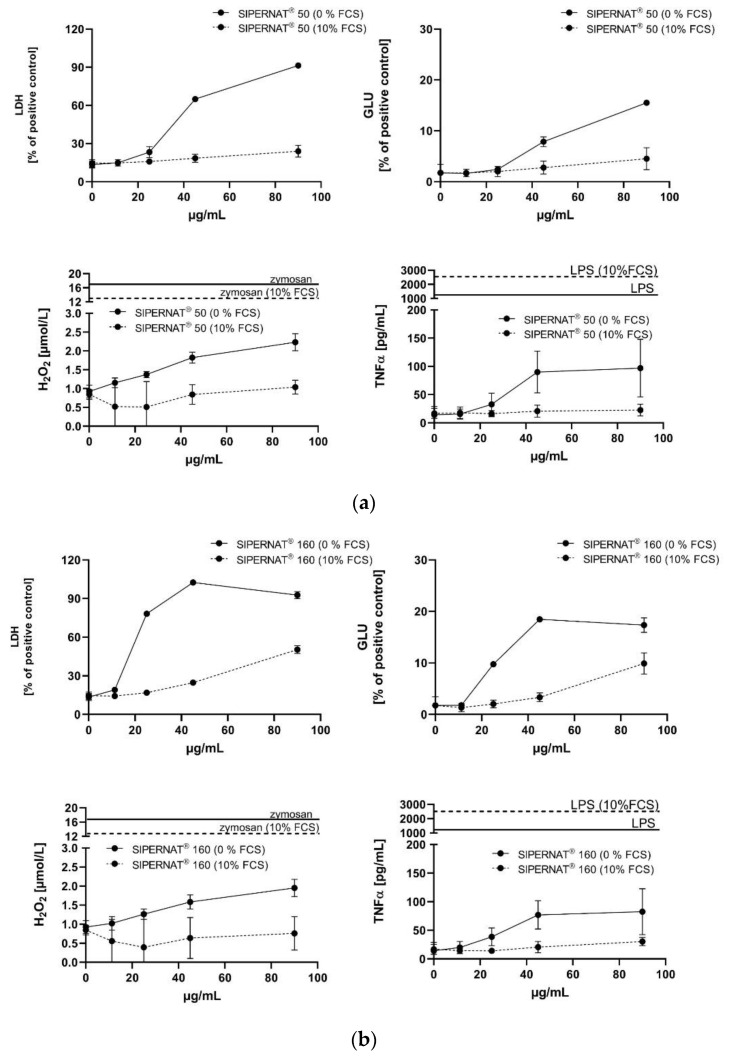
In vitro response of NR8383 alveolar macrophages to SIPERNAT^®^ 50 and SIPERNAT^®^ 160 in the absence or in the presence of FCS (dashed lines). Lactate dehydrogenase activity (LDH), glucuronidase activity (GLU), H_2_O_2_ concentration and tumour necrosis factor alpha (TNFα) were measured in the supernatant from NR8383 cells exposed to SIPERNAT^®^ 50 (**a**) or SIPERNAT^®^ 160 (**b**). Effects of zymosan and lipopolysaccharide (LPS) on the formation of H_2_O_2_ and TNFα, respectively, are indicated by vertical lines.

The biological activity of SIPERNAT^®^ 50 and SIPERNAT^®^ 160 was very similar to that of the AEROSIL^®^s (Figure 7a,b), though the dose-dependent release of LDH, GLU and TNFα was more pronounced for SIPERNAT^®^ 160. Again, 10% FCS abolished the H_2_O_2_ response and strongly reduced the cytotoxic effect (LDH, GLU) and also TNFα formation. There was a strong reduction of bioactivity in the presence of FCS. The shift in LOAEC upon FCS treatment are provided in Table 3. 

The electron microscopic examination revealed no major differences with respect to the endosomal compartments crowed by SIPERNAT^®^ 50 or SIPERNAT^®^ 160 particles. Even the fine structure of agglomerates within phagosomes appeared indistinguishable (Figures S1 and S2). With respect to SIPERNAT^®^ 160, endosomes with larger particle assemblies appeared less frequent (Figures S3 and S4). 

### 3.4. Evaluation of Data Using the Cell-Associated SiO_2_ Mass as a Dose Metric

Finally, we plotted the release of LDH, GLU and TNFα (Table S1) against the cell-associated particle mass. Except for AEROSIL^®^ 380 F, which partly adhered to the cell surface in the absence of FCS (Figure 3), the cell-associated particle mass in fact reflects fully internalized particles. Because a meaningful determination of SiO_2_ uptake relative to administered SAS concentration had to rely on non-compromised cells (i.e., at low cytotoxicity), we background corrected the “% total” values from Table 3 and extrapolated them to the maximum theoretical cell burden at a given concentration step (i.e., 7.5, 15, 30 and 60 pg/cell; see Supplementary Information for calculation). 

**Figure 8:**
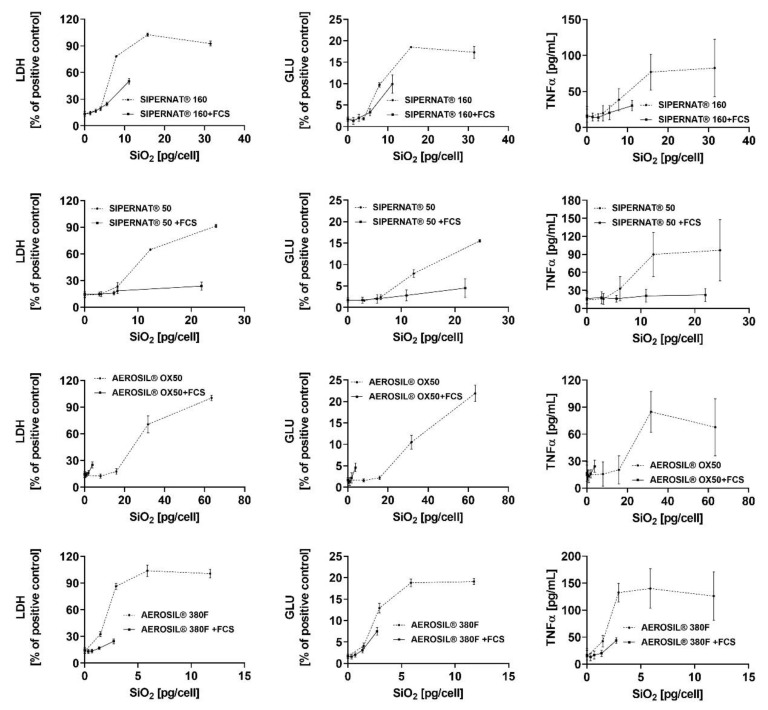
In vitro response of NR8383 alveolar macrophages to the cell-associated masses of SIPERNAT^®^ 160, SIPERNAT^®^ 50, AEROSIL^®^ OX50 and AEROSIL^®^ 380 F in the absence and presence of FCS. Values (from Table S1) for lactate dehydrogenase activity (LDH), glucuronidase activity (GLU), H_2_O_2_ concentration, and tumour necrosis factor alpha (TNFα) from were plotted against the cell-associated masses of SAS which were measured for a low SAS concentration and then extrapolated to higher values.

These uptake-corrected abscissa values were then plotted against the released enzyme activities the absence and presence of FCS (Figure 8). The FCS-mediated reduction in particle uptake is reflected by a shortening of the curves and the lowered slopes seen for the releases of LDH, GLU and TNFα indicate the FCS-mediated reduction of biological activity. The slope reductions (LDH, GLU and TNFα curves) appeared largely uniform for each singly SAS but differed in the order SIPERNAT^®^ 50 > SIPERNAT^®^ 160 = AEROSIL^®^ 380 F. AEROSIL^®^ OX50 was not evaluable due to a strong shortening of the respective curve. Resulting EC50 values were calculated for the FCS-free administration of SAS (Table S4) and will be discussed below. Overall, the curves shown in Figure 8 reveal that the reduced bioactivity of SAS in the presence of FCS is material dependent and due, at least in parts, to cellular processes secondary to particle uptake.

## 4. Discussion

In this investigation, we analysed the effect of FCS on the apparent bioactivity of fumed and precipitated SAS using an established alveolar macrophage model. For the first time, the uptake of SAS by alveolar macrophages was quantified with a high resolution ICP-MS technique. This enabled us to attribute the effect of FCS to both an influence on particle adhesion and subsequent uptake by cells, and an influence on the bioactivity of the cell-associated, i.e., ingested SAS material. 

The reduction of particle uptake in the presence of FCS was largest for both AEROSIL^®^s (69.5 to 83.5%): TEM analyses strongly suggest that the lower content of the AEROSIL^®^s in cells in the presence of FCS was mainly due to less particles captured within large phagosomes, whereas lysosomes or autophagosomes contained similar loads of particles under both conditions and, therefore, appear to be of minor relevance for the mitigating effect of FCS on the SAS effects. This appears to be different from the changing numbers of autophagosomes and lysosomes observed in alveolar macrophages treated with crystalline silica [40]. The failure to form larger particle-filled phagosomes may by explained, at least in part, by an absence of binding of AEROSIL^®^ 380 F to the cell surface in the presence of FCS. The lack of large particle-filled and afterwards disrupted phagosomes may have also prevented AEROSIL^®^ 380 F particles from entering into the cytoplasm under protein-free conditions, a mode of particle uptake strongly suggested by the patchy distribution pattern of SiO_2_ nanoparticles (Figure 3a). The reduced uptake of AEROSIL^®^ 380 F in the presence of FCS is seemingly in contrast to the strongly increased aggregate/agglomerate size (up to several hundred micrometers, see Table S1) and to the large number of precipitates visible with phase contrast optics (Figure 1a,b). We assume, however, that these particulates mainly consist of precipitated proteins which were cleared from the culture bottom most likely via ingestion by the NR8383 cells. Evidence for this assumption comes from numerous large vacuoles filled with low-contrast material possibly representing protein but only few silica particles (Figure 4a). Overall, the presence of FCS led to an enlargement of the AEROSIL^®^ 380 F particles’ HD, a reduced uptake into phagosomes, followed by an altered subcellular localization and reduced the biological activity. 

The influence of FCS on the uptake of both precipitated SAS was not uniform. While FCS reduced the uptake of SIPERNAT^®^ 160, most likely again by reducing the formation of particle-filled phagosomes, it had nearly no influence on the uptake of SIPERNAT^®^ 50. The lack of ultrastructural changes seen for SIPERNAT^®^ 50 upon FCS treatment was in line with the quantitative ICP-MS measurement. Therefore, the pronounced reduction of biologic activity seen for SIPERNAT^®^ 50 was more directly attributable to a change of biological particle properties. Of note, SIPERNAT^®^ 50 exhibited the smallest primary structures, the largest specific surface area, and also the highest number of reactive silanol groups as reflected by SEAR’s number (see Table 1). Reactive silanol groups of SAS are believed to contribute to the bioactivity of crystalline silica [41,42]. A protein corona, which will inevitably form around SAS in the presence of FCS [26,27], may keep biological structures at a distance from reactive structures at the SAS surface and this protective effect, as illustrated by the reduced slopes of LDH, GLU and TNFα release (Figure 8), may be larger for highly reactive SAS.

Interestingly, the reduction of biological activity by FCS found for ingested AEROSIL^®^ OX50 appeared to be solely due to the reduction in particle uptake (Figure 8). Unlike all other SAS nanomaterials, the slope of the shortened curve reflecting the effect of the ingested AEROSIL^®^ OX50 was not reduced, suggesting that FCS had a minor effect on the biologic activity of ingested AEROSIL^®^ OX50. Although a protein corona formation around this material is highly likely, its effect may be relatively small, possibly because AEROSIL^®^ OX50 had the smallest specific surface and the smallest SEAR’s number. Of note, the addition of protein to nanoparticles does not change or attenuate their biologic activity in general, as shown, e.g., for CeO_2_ [43], TiO_2_ or Fe_2_O_3_ [24]. Therefore, the specific surface reactivity of a given material needs to be taken into account if the effect of protein coating has to be predicted.

It may also be speculated that the primary particle size and/or the specific surface (BET value) correlates to the protective effect of FCS, because the highest reduction in bioactivity (80–90%) was found for AEROSIL^®^ 380 F (see Table 3). However, despite large differences in size and/or BET surface, the mitigating effects of FCS on all other SAS were highly similar (70–80%), arguing against a simple correlation of primary particles size and FCS-mediated reduction of bioactivity. On the other hand, there may be a size and/or surface-dependent influence of FCS on the uptake of SAS, which was more reduced for AEROSIL^®^ 380 F and also for the SIPERNAT^®^ 160 both of which exhibit the largest specific surface of the fumed and precipitated SAS, respectively (see Table 2: Ratio FCS-free/10% FCS). However, this effect may be indirectly caused via an influence of FCS on particle settling. 

In a previous test of the four SAS with the macrophage model, we found that the EC50 values for the release of LDH span a comparatively narrow range (from 13.2 µg/mL (AEROSIL^®^ 380 F) to 31.7 µg/mL (SIPERNAT^®^ 50). As shown here, this range becomes larger when the EC50 values are expressed as pg per cell (2.03 pg/cell (AEROSIL^®^ 380 F) to 28 pg/cell (AEROSIL^®^ OX50); see compilation in Table S4). The disparity of the EC50 values based on the cellular dose is likely to be more relevant because it is measured directly and is not deduced from particle agglomeration and settling. However, the knowledge of cell-associated particle burden helps not only to compare different experimental conditions. It is also urgently needed to better compare in vitro and in vivo data with the aim to refine in vitro tests by using adequate cellular doses. At present, in vivo experiments have mostly been evaluated for organ burden of nanoparticles. Values at the single cell level are rare, although some progress has been made, e.g., for silver laden phagocytes in lymph nodes, whose silver content has been estimated to reach up to 140 pg per cell [44].

The question of which type of protein coating in vitro adequately mimics the situation of particles in the lung is still unresolved. While it is beyond dispute that nanoparticles in body fluids such as blood or extracellular fluid carry a protein corona [13,43,45], protein corona formation in the lung parenchyma is more complex. At least in theory, a respirable (SAS) particle will first contact and adsorb biomolecules of the lung surfactant (phospholipids, various surfactant proteins) before it enters into the lung lining fluid with its multitude of different proteins [46,47,48]. During inhalation exposure, the dose rate is typically low and the binding of surfactant and protein components to inhaled particles may be complete and more or less well-structured. In contrast, the administration of a particle-containing fluid into the lung, i.e., a high dose rate, may locally disturb the lung’s surfactant layer and lead to unconventionally coated or even uncoated particles. In the case of colloidal SAS, this leads to a more intense inflammatory reaction of the rat lung during the first days after particle administration compared to inhalation exposure [36]. It is also noteworthy that in the case of an acute lung inflammation upon SAS, the protein concentration of the lung lining fluid will rise [33], which may limit the bioactivity of SAS as observed in vitro. 

Based on these considerations and on the findings of this study, the way of in vitro testing being most predictive for the in vivo outcome remains a matter of discussion. Depending on the starting conditions, alveolar macrophages in vivo may engulf uncoated as well as protein-coated particles. However, we suggest that the well-established alveolar macrophage assay with NR8383 cells, which was originally developed and validated as a protein-free approach [32] and, as such, has been successfully incorporated into a tiered grouping strategy for nanomaterials [49], may be expanded for effects of proteins on the in vitro bioactivity of nanomaterials. As shown here for SAS, protein-free and protein-supplemented exposure of cells may differ substantially and the effects of both treatments should be understood as corner points possibly spanning the full range of responses of alveolar macrophages in situ. In any case, the additional inclusion of protein-containing assays needs to be supported by a quantification of nanomaterials’ uptake to avoid unwarranted conclusions. To this end, the method introduced here is applied as a reliable tool to quantify SAS nanomaterials at the cell culture level.

## Figures and Tables

**Table 1:** Material properties of the synthetic amorphous silica (SAS) used in the study.

Material	Size of Primary Structures (nm) ^(1)^	Aggregate Size (nm) ^(1)^	BET (m^2^/g) ^(2)^	Zeta Potential (mV) ^(3)^	Solubility (mg/L) ^(4)^	pH ^(5)^	SEARs No. ^(6)^
**AEROSIL^®^ 380F**	8.0 ± 2.7	101.9	390	−36	226.0	4.2	14.5
**AEROSIL^®^ OX50**	41.4 ± 18.3	233.7	45	−40	117.9	4.6	1.8
**SIPERNAT^®^ 50**	3.1. ± 0.7	59.8	460	−21	113.9	6.3	16.3
**SIPERNAT^®^ 160**	12.2. ± 2.7	58.3	180	−53	112.1	6.1	11.1

^(1)^ As measured by transmission electron microscopy. ^(2)^ Specific surface area measured by N_2_ adsorption. ^(3)^ Zeta potential and point of zero charge. ^(4)^ Measured according to enhanced OECD 105 Test Guideline on solubility. ^(5)^ Measured in 5% solution; ^(6)^ a measure for the number of silanol groups on the surface of silica according. For details, see [18].

**Table 2:** Quantification of SiO_2_ uptake in the absence and presence of foetal calf serum (FCS).

	10% FCS		FCS-Free		
Material	Cell-Associated SiO_2_ (µg) ^(1)^	% Total ^(2)^	Cell-Associated SiO_2_ (µg) ^(1)^	% Total ^(2)^	Ratio FCS-Free/10% FCS ^(3)^
**AEROSIL^®^ 380 F**	4.4/4.4	6.6/6.6	13.3/15.6	19.9/23.4	3.28
**AEROSIL^®^ OX50**	5.7/5.8	8.4/8.6	33.3/37.8	49.5/56.2	6.18
**SIPERNAT^®^ 50**	25.5/26.7	42.3/44.3	27.8/30.1	46.1/49.9	1.11
**SIPERNAT^®^ 160**	13.3/14.4	21.1/22.8	34.4/38.9	54.5/61.6	2.65
**Control ^(4)^**	1.2/1.6	-/-	1.2/1.3	-/-	-

^(1)^ Amounts of SiO_2_ in the cell pellet after 16 h, as measured by ICP-MS; values measured in duplicates are separated by a slash. ^(2)^ Cell-associated SiO_2_ in percent of the total added mass (67.5 µg SAS per 6 mL medium; weigh in of each SAS was individually corrected for its water content measured as loss on ignition [18]. ^(3)^ Ratios were calculated from mean values of columns 2 and 4. ^(4) 28^Si values from control cells were assumed to represent SiO_2_ and were converted equivalently.

**Table 3:** Apparent reduction of the bioactivity of SAS nanoparticles upon addition of FCS.

				Low Observed Adverse Effect Concentration (LOAEC) Shift (µg/mL) ^(2)^			
Material	LDH ^(1)^	GLU ^(1)^	TNFα ^(1)^	LDH	GLU	H_2_O_2_	TNFα
**AEROSIL^®^ 380F**	−92.6%	−81.2%	−87.4%	11.25⟶90	11.25⟶90	90⟶≥90	45⟶≥90
**AEROSIL^®^ OX50**	−68.9%	−65.9%	−81.2%	45⟶90	45⟶90	90⟶≥90	45⟶≥90
**SIPERNAT^®^ 50**	−72.3%	−64.1%	−81.9%	22.5⟶90	45⟶90	45⟶≥90	45⟶≥90
**SIPERNAT^®^ 160**	−79.6%	−71.6%	−79.6%	11.25⟶45	22.5⟶90	90⟶≥90	45⟶≥90

^(^^1)^ Measured at 90 µg/mL by linear interpolation; ^(^^2)^ LOAEC with and without FCS as derived from Table S3. LDH: Lactate dehydrogenase, GLU: glucuronidase, TNFα: tumor necrosis factor α.

## Data Availability

The data presented in this study are available in the article or in the Supplementary Material.

## Supplementary Materials

The following are available online at https://www.mdpi.com/2079-4991/11/3/628/s1, Figure S1. Electron microscopy of NR8383 cells laden with SIPERNAT^®^ 50 in the absence of FCS for 16 h. Figure S2. Electron microscopy of NR8383 cells laden with SIPERNAT^®^ 50 in the presence of FCS for 16 h. Figure S3. Electron microscopy of NR8383 cells laden with SIPERNAT^®^ 160 in the absence of FCS for 16 h. Figure S4. Electron microscopy of NR8383 cells laden with SIPERNAT^®^ 160 in the presence of FCS for 16 h. Table S1. Hydrodynamic diameter of SAS in H2O and cell culture media. Table S2. Trypan Blue Exclusion Test and cell numbers of SAS-treated cells used for mass spectrometric. Table S3. Numerical values from in vitro tests with the Alveolar Macrophage Model. Table S4. EC50 values for cell-associated SAS causing the release of LDH, GLU and TNFα. Table S5. EC50 values calculated for extrapolated intracellular SAS concentrations.
