# Supplementary material for: Serum Lowers Bioactivity and Uptake of Synthetic Amorphous Silica by Alveolar Macrophages in a Particle Specific Manner
Source: Nanomaterials (Basel). 2021 Mar 3;11(3):628. doi: 10.3390/nano11030628 (PMC7999370; doi:10.3390/nano11030628)
Supplement: Supplementary file 1 [file nanomaterials-11-00628-s001.pdf]

nanomaterials

Supplementary Materials

Serum Lowers Bioactivity and Uptake of Synthetic Amorphous Silica by Alveolar Macrophages in a Particle Specific Manner

Martin Wiemann [1,*], Antje Vennemann [1], Cornel Venzago [2], Gottlieb-Georg Lindner [3], Tobias B. Schuster [2] and Nils Krueger [2]

1 IBE R&D Institute for Lung Health gGmbH, Mendelstr. 11, D-48149 Münster, Germany; vennemann@ibe-ms.de
2 Evonik Operations GmbH, Rodenbacher Chaussee 4, 63457 Hanau-Wolfgang, Germany; cornel.venzago@evonik.com (C.V.); tobias.schuster@evonik.com (T.B.S.); nils.krueger@evonik.com (N.K.)
3 Evonik Operations GmbH, Brühler Straße 2, 50389, Wesseling, Germany; gottlieb-georg.lindner@evonik.com (G.-G.L.)
* Correspondence: martin.wiemann@ibe-ms.de; Tel.: +49 251 9802340

Calculation of Mean Cellular Particle Burden as used in Figure 8

To calculate the mean cellular burden under the conditions of the macrophage model the totally applied dose of particles per well of a 96-well plate is calculated from the particle concentration per mL multiplied by 0.2, divided by the initial number of cells in the vial, which is 3×10^5 cells/well und serum-free conditions. Provided that the material is taken up completely by the cells, the concentration range of 11.25, 22.5, 45 and 90 µg/mL, may then be converted to a mean cellular burden of 7.5, 15, 30 and 60 pg/cell, respectively. To obtain the cellular burden shown in Figure 8 (abscissa values) each mean cellular burden was multiplied by the percentage of ingested particles found for the lowest concentration only. Importantly, this concentration was non-toxic (10% FCS added) or nearly non-toxic (protein-free). A direct measurement of the particle load over the whole concentration range would have caused progressive cell lysis, counteracting the isolation of particle-laden cells and subsequent measurements of cell-associated silica. The calculation used here circumvents this problem but should be understood as an approximation.

Table S1. Hydrodynamic diameter of SAS in H_2O and cell culture media.

Material	Fluid	% FCS	Hydrodynamic Diameter [nm]				
			Mean ± SEM	Mode ± SEM	d10 ± SEM	d50 ± SEM	d90 ± SEM
**AEROSIL® 380 F**	H_2O	0	n.d.	n.d.	n.d.	n.d.	n.d.
	KRPG	0	151.7 ± 0.6	104.0 ± 2.7	90.3 ± 1.5	135.7 ± 0.4	223.8 ± 3.2
	KRPG	10	163.2 ± 1.5	112.8 ± 1.3	99.5 ± 0.7	132.3 ± 1.7	256.7 ± 3.0
	F-12K	0	168.5 ± 2.3	134.2 ± 9.6	110.2 ± 2.2	154.3 ± 2.1	230.8 ± 7.3
	F-12K	10	436.3 ± 5.4	457.7 ± 30.3	247.6 ± 6.8	442.0 ± 9.6	593.3 ± 14.1
**AEROSIL® OX50**	H_2O	0	184.0 ± 1.8	178.1 ± 7.2	122.5 ± 1.2	170.9 ± 2.4	247.2 ± 5.1
	KRPG	0	367.5 ± 12.4	365.5 ± 37.6	218.9 ± 14.8	363.2 ± 13.8	493.9 ± 5.7
	KRPG	10	214.8 ± 0.6	200.6 ± 12.8	140.8 ± 1.0	201.0 ± 3.9	287.5 ± 2.8
	F-12K	0	198.4 ± 0.6	159.7 ± 5.0	135.3 ± 0.7	182.8 ± 0.7	263.8 ± 1.3
	F-12K	10	217.8 ± 2.2	190.0 ± 8.0	145.9 ± 4.5	202.9 ± 1.2	290.6 ± 2.4
**SIPERNAT® 50**	H_2O	0	238.7 ± 3.3	172.4 ± 2.3	147.2 ± 2.1	200.9 ± 4.1	373.3 ± 6.8
	KRPG	0	227.6 ± 5.5	174.9 ± 3.4	138.4 ± 3.5	190.0 ± 5.5	363.2 ± 11.4
	KRPG	10	248.0 ± 0.9	181.9 ± 7.3	157.0 ± 3.8	212.8 ± 2.2	378.7 ± 0.6
	F-12K	0	251.3 ± 0.7	164.7 ± 0.3	146.6 ± 1.2	217.5 ± 4.5	410.1 ± 12.7
	F-12K	10	258.2 ± 2.6	185.1 ± 6.8	154.4 ± 2.4	216.9 ± 4.3	419.6 ± 2.5
**SIPERNAT® 160**	H_2O	0	212.5 ± 0.8	146.4 ± 3.4	127.6 ± 0.7	180.5 ± 2.1	325.8 ± 2.0
	KRPG	0	269.2 ± 11.1	188.1 ± 3.6	165.5 ± 6.1	241.3 ± 11.7	415.4 ± 18.0
	KRPG	10	276.9 ± 6.0	194.7 ± 10.0	165.5 ± 3.2	248.5 ± 2.9	421.1 ± 6.8
	F-12K	0	226.2 ± 5.3	173.7 ± 11.0	138.6 ± 1.8	194.6 ± 5.6	344.4 ± 14.6
	F-12K	10	235.6 ± 3.9	182.2 ± 5.5	141.0 ± 2.5	202.5 ± 3.7	356.7 ± 12.6

Footnote to Table S1. Values for d10, d50, d90 describe the cumulative particle size distribution at 10%, 50% and 90% of the maximum value. Particle concentration was 9 µg/mL in all measurements. n.d.: particles not detectable.

Table S2. Trypan Blue exclusion test and cell numbers of SAS-treated cells used for mass spectrometric determination of the cell-associated SiO_2 mass.

Material	Effects of SAS Treatment			
	Serum-free		10 % Serum	
	cells/mL	% dead cells	cells/mL	% dead cells
Control Cells	345,000	3.6	490,000	1.5
SIPERNAT® 160	420,000	9.4	632,500	1.2
SIPERNAT® 50	480,000	7.3	630,000	2.4
AEROSIL® OX50	400,000	4.2	592,500	2.5
AEROSIL® 380 F	447,500	10.6	662,500	1.5

Footnote to Table S2. Values are means from values measured in duplicates.

Table S3. Numerical values from in vitro tests with the Alveolar Macrophage Model.

		LDH [% pos. Control]		GLU [% pos. Control]		H_2O_2 [µg/mL]		TNFα [pg/mL]	
**Material**	**[µg/mL]**	**w/o FCS**	**10 % FCS**	**w/o FCS**	**10 % FCS**	**w/o FCS**	**10 % FCS**	**w/o FCS**	**10 % FCS**
**AEROSIL® 380 F**	**0**	13.4 ± 2.7	14.5 ± 3.0	1.7 ± 0.5	1.7 ± 1.7	0.9 ± 0.2	0.8 ± 0.1	14.3 ± 14.7	16.7 ± 8.9
	**11.25**	32.5 ± 2.9 ***	13.2 ± 2.3	4.1 ± 0.7 *	1.6 ± 0.6	1.2 ± 0.1	0.8 ± 0.2	42.5 ± 11.1	13.1 ± 7.0
	**22.5**	86.4 ± 3.2 ***	13.5 ± 1.9	12.9 ± 1.1 ***	2.0 ± 0.5	1.2 ± 0.1	0.6 ± 0.5	132.5 ± 17.2 ***	17.0 ± 7.3
	**45**	103.9 ± 6.4 ***	16.8 ± 1.5	18.8 ± 0.9 ***	3.1 ± 0.7	1.4 ± 0.1	0.9 ± 0.2	140.1 ± 36.7 ***	20.0 ± 5.8
	**90**	100.8 ± 5.1 ***	24.5 ± 2.8 **	19.1 ± 0.7 ***	7.5 ± 0.9 ***	1.9 ± 0.2 ***	0.9 ± 0.2	126.1 ± 44.7 ***	43.7 ± 5.0
**AEROSIL® OX50**	**0**	13.4 ± 2.7	14.5 ± 3.0	1.7 ± 0.5	1.7 ± 1.7	0.9 ± 0.2	0.8 ± 0.1	14.3 ± 14.7	16.7 ± 8.9
	**11.25**	12.6 ± 2.3	13.0 ± 2.2	1.6 ± 0.4	1.4 ± 0.6	1.0 ± 0.2	0.9 ± 0.1	15.6 ± 13.5	14.6 ± 6.1
	**22.5**	17.9 ± 3.2	14.6 ± 2.4	2.2 ± 0.4	1.3 ± 0.9	1.2 ± 0.0	1.0 ± 0.2	20.3 ± 15.7	13.0 ± 7.0
	**45**	70.8 ± 9.5 ***	16.1 ± 2.6	10.5 ± 1.6 ***	2.2 ± 1.2	1.2 ± 0.1	0.9 ± 0.3	84.9 ± 22.6 ***	15.9 ± 4.6
	**90**	100.4 ± 3.0 ***	25.2 ± 3.4 *	21.9 ± 1.9 ***	4.6 ± 1.0 *	1.7 ± 0.2 ***	0.9 ± 0.2	67.8 ± 31.7 **	24.4 ± 6.8
**SIPERNAT® 50**	**0**	13.4 ± 2.7	14.5 ± 3.0	1.7 ± 0.5	1.7 ± 1.7	0.9 ± 0.2	0.8 ± 0.1	14.3 ± 14.7	16.7 ± 8.9
	**11.25**	14.9 ± 2.7	14.8 ± 2.3	1.6 ± 0.2	1.7 ± 0.7	1.2 ± 0.1	0.5 ± 0.6	15.8 ± 8.9	17.9 ± 10.3
	**22.5**	23.3 ± 4.4 **	15.9 ± 2.3	2.4 ± 0.6	2.0 ± 1.0	1.4 ± 0.1	0.5 ± 0.7	32.9 ± 19.9	16.3 ± 5.4
	**45**	64.9 ± 1.1 ***	18.5 ± 3.2	7.9 ± 0.9 ***	2.8 ± 1.3	1.8 ± 0.1 *	0.8 ± 0.3	90.0 ± 36.8 **	20.9 ± 10.6
	**90**	91.4 ± 1.9 ***	23.9 ± 4.6 **	15.5 ± 0.3 ***	4.5 ± 2.2 *	2.2 ± 0.2 ***	1.0 ± 0.2	97.0 ± 51.1 ***	22.5 ± 10.7
**SIPERNAT® 160**	**0**	13.4 ± 2.7	14.5 ± 3.0	1.7 ± 0.5	1.7 ± 1.7	0.9 ± 0.2	0.8 ± 0.1	14.3 ± 14.7	16.7 ± 8.9
	**11.25**	19.1 ± 2.1 *	14.3 ± 2.1	1.8 ± 0.3	1.3 ± 0.8	1.0 ± 0.2	0.6 ± 0.6	19.9 ± 10.7	14.6 ± 5.5
	**22.5**	78.2 ± 1.0 ***	16.8 ± 2.0	9.7 ± 0.5 ***	2.0 ± 0.8	1.3 ± 0.1	0.4 ± 0.9	38.5 ± 15.5	14.0 ± 5.0
	**45**	102.5 ± 1.7 ***	24.7 ± 2.3 ***	18.5 ± 0.2 ***	3.3 ± 0.8	1.6 ± 0.2	0.6 ± 0.5	77.0 ± 24.8 **	20.7 ± 9.9
	**90**	92.6 2.7 ***	50.3 3.1 ***	17.3 ± 1.4 ***	9.9 ± 2.1 ***	1.9 ± 0.2 *	0.8 ± 0.4	82.5 ± 40.1 ***	30.3 ± 7.2
**Corundum**	**0**	13.2 ± 2.3	16.1 ± 2.9	1.7 ± 0.4	1.7 ± 0.4	1.1 ± 0.3	0.9 ± 0.1	15.3 ± 11.9	16.5 ± 6.8
	**22.5**	13.6 ± 2.5	12.9 ± 1.9	2.1 ± 0.7	1.5 ± 2.3	1.1 ± 0.2	0.9 ± 0.1	11.6 ± 7.8	12.8 ± 8.4
	**45**	15.1 ± 3.4	15.3 ± 1.9	1.8 ± 0.6	1.5 ± 2.1	1.2 ± 0.2	1.0 ± 0.1	12.2 ± 7.1	12.9 ± 7.4
	**90**	18.0 ± 3.3	17.2 ± 1.5	2.0 ± 1.0	2.2 ± 1.5	1.1 ± 0.3	1.0 ± 0.1	12.9 ± 10.0	14.3 ± 8.4
	**180**	21.5 ± 2.9 *	18.9 ± 1.3	2.6 ± 0.8	3.4 ± 1.0	1.7 ± 0.3 **	1.2 ± 0.1	15.2 ± 13.4	11.9 ± 6.1
**Quartz DQ12**	**0**	13.2 ± 2.3	16.1 ± 2.9	1.7 ± 0.4	1.7 ± 0.4	1.1 ± 0.3	0.9 ± 0.1	15.3 ± 11.9	16.5 ± 6.8
	**22.5**	12.3 ± 2.2	13.3 ± 0.9	1.8 ± 0.3	2.3 ± 1.4	1.1 ± 0.3	0.9 ± 0.1	12.8 ± 8.2	14.2 ± 7.8
	**45**	15.8 ± 3.1	15.4 ± 1.3	2.0 ± 0.6	3.2 ± 1.9	1.2 ± 0.3	0.9 ± 0.1	13.7 ± 10.6	14.4 ± 4.1
	**90**	34.6 ± 7.8 ***	28.9 ± 4.2 ***	4.4 ± 1.6	7.2 ± 2.7 **	0.9 ± 0.4	1.0 ± 0.2	38.8 ± 25.8	34.6 ± 8.8
	**180**	78.7 ± 9.9 ***	61.9 ± 5.6 ***	15.4 ± 4.2 ***	20.3 ± 3.5 ***	2.0 ± 0.3 ***	1.1 ± 0.2	101.2 ± 35.9 ***	99.1 ± 16.8 ***
**Zymosan**	**360**	-	-	-	-	15.2 ± 0.4 ***	11.6 ± 0.7 ***	-	-
**LPS**	**0.5**	-	-	-	-	-	-	1153 ± 214 ***	2634 ± 55.4 ***

Footnote to Table S3. LDH: lactate dehydrogenase, GLU: glucuronidase, H_2O_2: hydrogene peroxide, TNF: tumor necrosis factor α, LPS: lipopolysaccharide. * Level of significance is shown by asterisks with *: $P < 0.05$, **: $P < 0.01$, and ***: $P < 0.001$.

Table S4. EC50 values for the release of LDH, GLU and TNFα calculated for cell-associated SAS.

Particle Name	LDH		GLU		TNFα	
	**EC50 [pg/cell]**	**Goodness of Fit (R2)**	**EC50 [pg/ cell]**	**Goodness of Fit (R2)**	**EC50 [pg/cell]**	**Goodness of Fit (R2)**
SIPERNAT® 160	6.38 *(15.5)*	0.992	7.87 *(19.4)*	0.997	9.26	0.998
SIPERNAT® 50	10.60 *(31.7)*	0.998	13.61*(36.3)*	0.993	7.86	1.000
AEROSIL® OX50	28.00 *(22.7)*	1.000	33.61*(36.5)*	0.999	17.16	0.967
AEROSIL® 380F	2.03 *(13.2)*	0.998	2.50 *(14.0)*	1.000	1.67	0.993

Footnote to Table S4. Values were non-linear fittings from the curves shown in Figure 8 using GraphPad Prism 8 Software. Italic values in brackets are EC50 values previously determined in μg/mL [18].

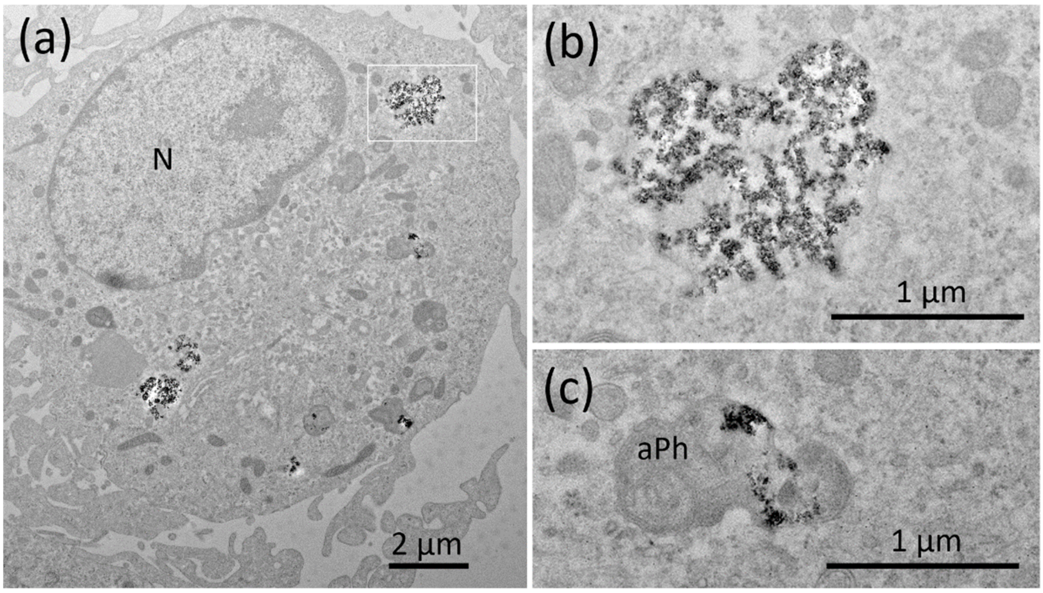

Figure S1. Electron microscopy of NR8383 cells laden with SIPERNAT® 50 in the absence of FCS for 16 h. (**a**) Overview of a cell with particle-containing phagosomes (large arrows), autophagosomes (aPh) and lysosomes; N: nucleus; the white box is enlarged in (**b**) and shows a particle-filled phagosome; (**c**) an autophagosomes with condensed particulates (aPh).

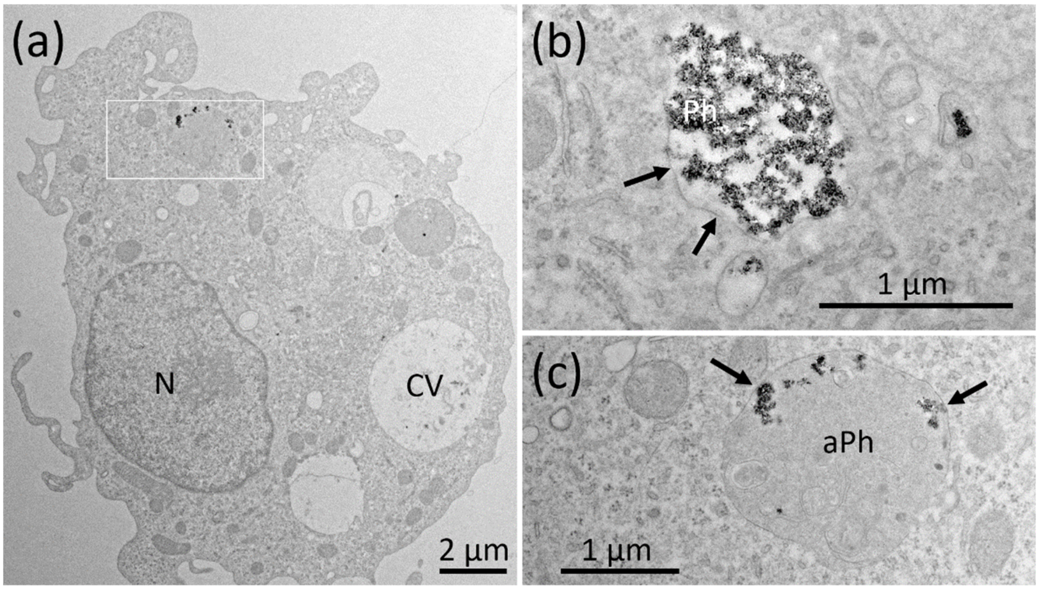

Figure S2. Electron microscopy of NR8383 cells laden with SIPERNAT® 50 in the presence of FCS for 16 h. (**a**) Overview of a cell with particle-containing autophagosome; CV: clear vesicle; N: nucleus. (**b**) particle-filled phagosome (Ph); the white box in (**a**) is enlarged in (**c**) and shows an autophagosome (aPh) with condense particulates. Arrows in (**b**) and (**c**) point to continuous membranes of the particle-containing vesicles.

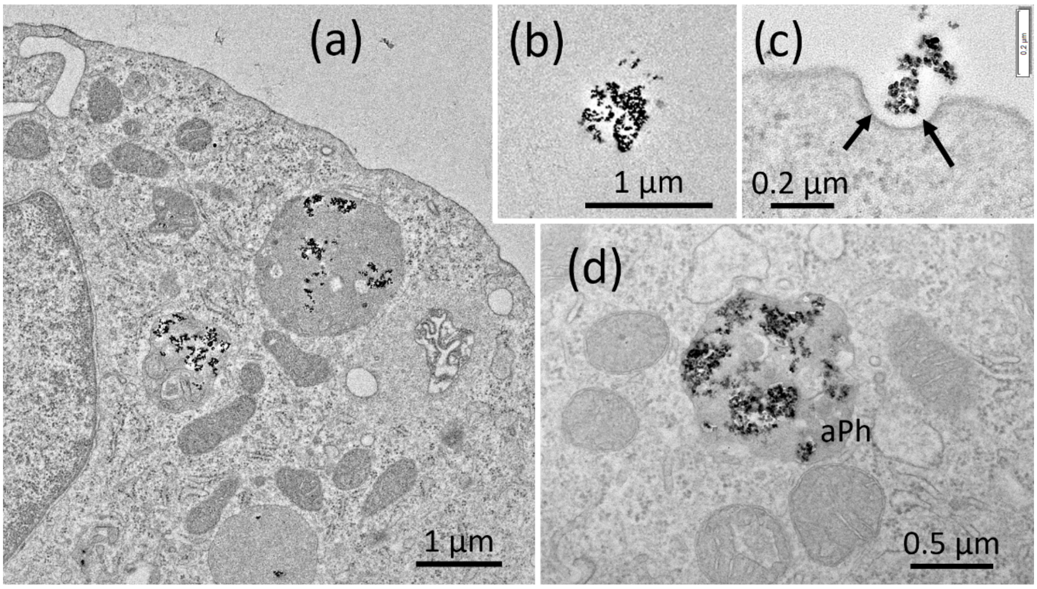

Figure S3. Electron microscopy of NR8383 cells laden with SIPERNAT® 160 in the absence of FCS for 16 h. (**a**) Section of a cell with particle-containing vesicles. Extracellular aggregates/agglomerates of SIPERNAT® 160 in the medium (**b**), and (**c**) close to the cell membrane during beginning endosome formation (large arrows). (**d**)Autophagosome (aPh) partly filled with particles aggregates/agglomerates.

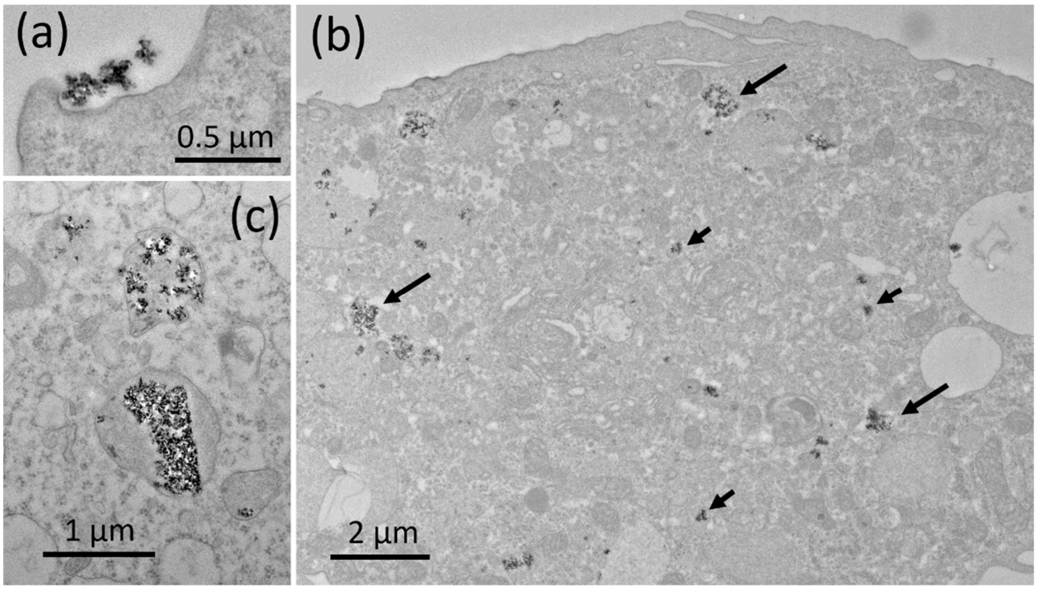

Figure S4. Electron microscopy of NR8383 cells laden with SIPERNAT® 160 in the presence of FCS for 16 h. (**a**) Extracellular aggregates/agglomerates of SIPERNAT® 160 close to the cell membrane during early endosome formation. (**b**) Section of a cell with particle-containing phagosomes (large arrows) and numerous small particle deposits in endosomes (small arrows), lysosomes and autophagosomes. (**c**) Aggregates/agglomerates of SIPERNAT® 160 lysosomes.
